# Predicting compressive strength of RCFST columns under different loading scenarios using machine learning optimization

**DOI:** 10.1038/s41598-023-43463-6

**Published:** 2023-10-03

**Authors:** Feng Wu, Fei Tang, Ruichen Lu, Ming Cheng

**Affiliations:** 1 School of Architectural Engineering, Xinyang Vocational and Technical College, Xinyang, 464000 China; 2 China Construction Fifth Engineering Division Corp., Ltd., Changsha, 410000 China

**Keywords:** Energy science and technology, Engineering, Materials science, Mathematics and computing

## Abstract

Accurate bearing capacity assessment under load conditions is essential for the design of concrete-filled steel tube (CFST) columns. This paper presents an optimization-based machine learning method to estimate the ultimate compressive strength of rectangular concrete-filled steel tube (RCFST) columns. A hybrid model, GS-SVR, was developed based on support vector machine regression (SVR) optimized by the grid search (GS) algorithm. The model was built based on a sample of 1003 axially loaded and 401 eccentrically loaded test data sets. The predictive performance of the proposed model is compared with two commonly used machine learning models and two design codes. The results obtained for the axial loading dataset with R^2^ of 0.983, MAE of 177.062, RMSE of 240.963, and MAPE of 12.209%, and for the eccentric loading dataset with R^2^ of 0.984, MAE of 93.234, RMSE of 124.924, and MAPE of 10.032% show that GS-SVR is the best model for predicting the compressive strength of RCFST columns under axial and eccentric loadings. It is an effective alternative method that can be used to assist and guide the design of RCFST columns to save time and cost of some laboratory experiments. Additionally, the impact of input parameters on the output was investigated.

## Introduction

An infill component known as a "concrete-filled steel tube" (CFST) is a structural system consisting of an outer steel tube and a core filled with concrete^1^. The most commonly used types of CFST columns are circular concrete-filled steel tube (CCFST) and rectangular concrete-filled steel tube (RCFST), which effectively utilize the complementary action between concrete and steel. Compared to conventional reinforced concrete or pure steel elements, the CFST system provides mechanical advantages due to the steel tube's restraining effect on the filled concrete, substantially improving ductility and strength^2–7^. Additionally, the concrete core restrains the inward deformation of the steel tube, retarding local buckling and enhancing overall column stability^8,9^. These synergistic effects lead to increased strength and performance characteristics over the respective individual parts. Due to their high strength, resilience, effective seismic energy absorption, and excellent fire resistance, CFST columns are commonly used in high-rise buildings, bridges, and other infrastructure projects^10,11^ .

The primary mechanical characteristic of CFST is its compressive strength, which plays a critical role in the accurate design of CFST columns to ensure structural stability. To better understand the behavior of CFST under loading, researchers commonly use experimental and finite element methods to estimate their performance^12,13^. Physical experiments can provide valuable insights, but they are resource-intensive and time-consuming. On the other hand, finite element analysis can reduce the number of tests required through computer simulation, but its accuracy depends heavily on the expertise of the modeler and requires high computer configurations. To address these limitations, some countries have developed equation-based design standards, such as ACI 318 (ACI 2014), Eurocode 4 (CEN 2004), AISC 360-16 (AISC 2016), and Chinese codes (GB 50936-2014 and GB/T 51446-2021), which are based on extensive experimental results. Using design codes to predict compressive strength is currently a more practical option than physical experiments or finite element analysis. However, it is important to note that these empirical formulas have their corresponding scope of application and may not be suitable for all CFST columns, which vary in material strength, shape, cross-sectional length, and slenderness. Therefore, using these standards to calculate the strength of CFST columns may carry a certain level of risk, and additional caution and analysis may be required.

Machine learning techniques have the potential to provide accurate and efficient predictions of the bearing capacity of CFST components^14–18^. These methods can utilize large volumes of experimental data to identify patterns and relationships that are difficult to detect using traditional methods. Some of the machine learning methods that have been used for this purpose include artificial neural networks (ANN), gene expression programming (GEP), back-propagation neural networks (BPNN), and fuzzy logic. By leveraging these techniques, researchers have been able to successfully predict the carrying capacity of CFST, which can provide valuable insights for designing structures and reducing the need for further testing. Overall, the application of machine learning to CFST design represents an exciting and promising area of research^19–28^. In order to implement the ultimate compressive strength prediction of RCFST columns, Mai et al.^29^ developed an ANN network that was optimized by the particle swarm optimization algorithm. The results revealed that the proposed hybrid model has higher prediction accuracy than the traditional design codes. The BAS-MLP model was created by Ren et al.^30^ using a multilayer perceptron (MLP) neural network coupled with a beetle antenna search (BAS) algorithm to forecast the ultimate bearing capacity of RCFST columns. The outcomes demonstrated that the BAS-MLP model performs better than a number of benchmark models and traditional approaches. To forecast the maximum load capacity of short rectangular columns of restrained reinforced concrete (SCFST), Lu et al.^31^ established a predictive method based on the gradient boost regression tree (GBRT) model. The results of a straightforward comparison of many regression models revealed that the GBRT model makes a fair prediction of the mechanical characteristics of SCFST columns. The ANN-PSO model was used by Kim et al.^29,32^ to forecast the eccentric load capacity of 241 CCFST columns and 622 RCFST columns, respectively. The findings revealed that the average prediction errors were 12.1% and 15.4%, respectively, which is better than the traditional design codes. On the basis of 1224 test data, Panagiotis et al.^33^ developed an ANN model for the ultimate compressive capacity of RCFST columns with seven variables, including the column's width and height, steel tube thickness, effective length, steel yield strength, concrete compressive strength, and eccentricity. They then compared the developed model with the design codes currently in use. It was revealed that its accuracy was greatly enhanced while keeping the forecast findings steady. Also, an explicit equation is provided for simple implementation and use evaluation. Quang et al.^34^ developed a gradient tree boosting approach to forecast the strength of the CFST column, and the proposed model produced higher prediction accuracy when compared to deep learning, decision trees, random forests, and support vector machines (SVM).

Research on predicting the strength of CFST columns using machine learning seems to have made some progress. However, most studies have focused on using traditional machine learning models to forecast the axial compression strength of CFST columns. These models are limited by the selection of hyper-parameters, resulting in restricted prediction accuracy. Optimized hybrid models have the potential to improve prediction performance, but there is limited research in this area and further studies are necessary. Furthermore, current research primarily focuses on load-bearing capacity predictions, with less emphasis on the feature importance analysis of design parameters, which is particularly valuable for CFST design. To achieve this objective, this study aims to establish an optimization model for the compressive strength of RCFST under axial and eccentric loading conditions and analyze the impact of these design parameters on the output results.

**Figure 1:**
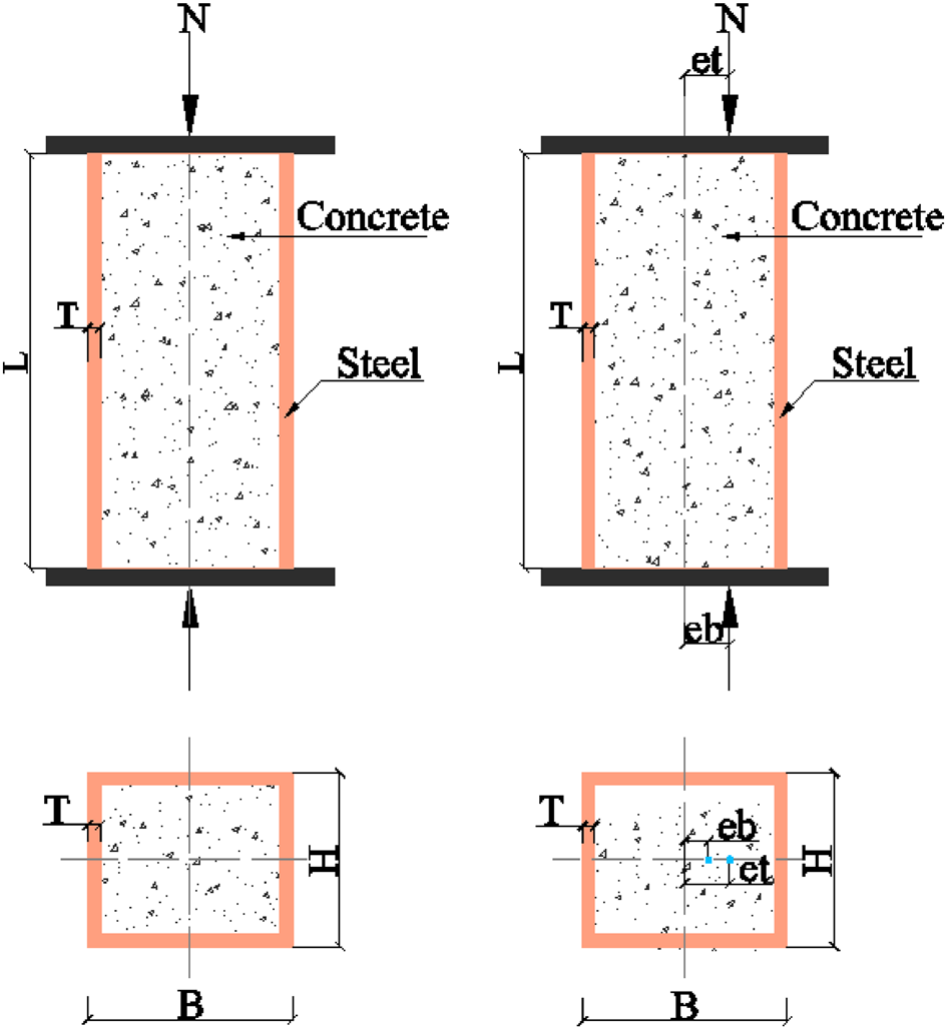
Schematic diagram of RCFST columns under axial and eccentric loading.

As shown in Fig. 1, the input parameters consist of both geometric features and material properties. For RCFST, the specific input variables include column width (B), height (H), thickness (T), length (L), yield strength (fy), compressive strength (fc), top eccentricity (et), and bottom eccentricity (eb). The performance of the proposed optimization model was compared with that of conventional support vector regression (SVR) and random forest (RF) models to determine the optimal prediction model for this research. Moreover, the Shapley additive explanations (Shap) analysis method has been introduced to assess the roles and impacts of these design parameters.

## Methodology

### Random forest model

The random forest algorithm is a machine learning method that combines decision trees, random feature selection, and integration to create a powerful combinatorial classifier. It uses a self-help approach to perform bootstrap sampling and generate training subsets, ensuring that *n* random samples produce *n* training sets of the same size.

**Figure 2:**
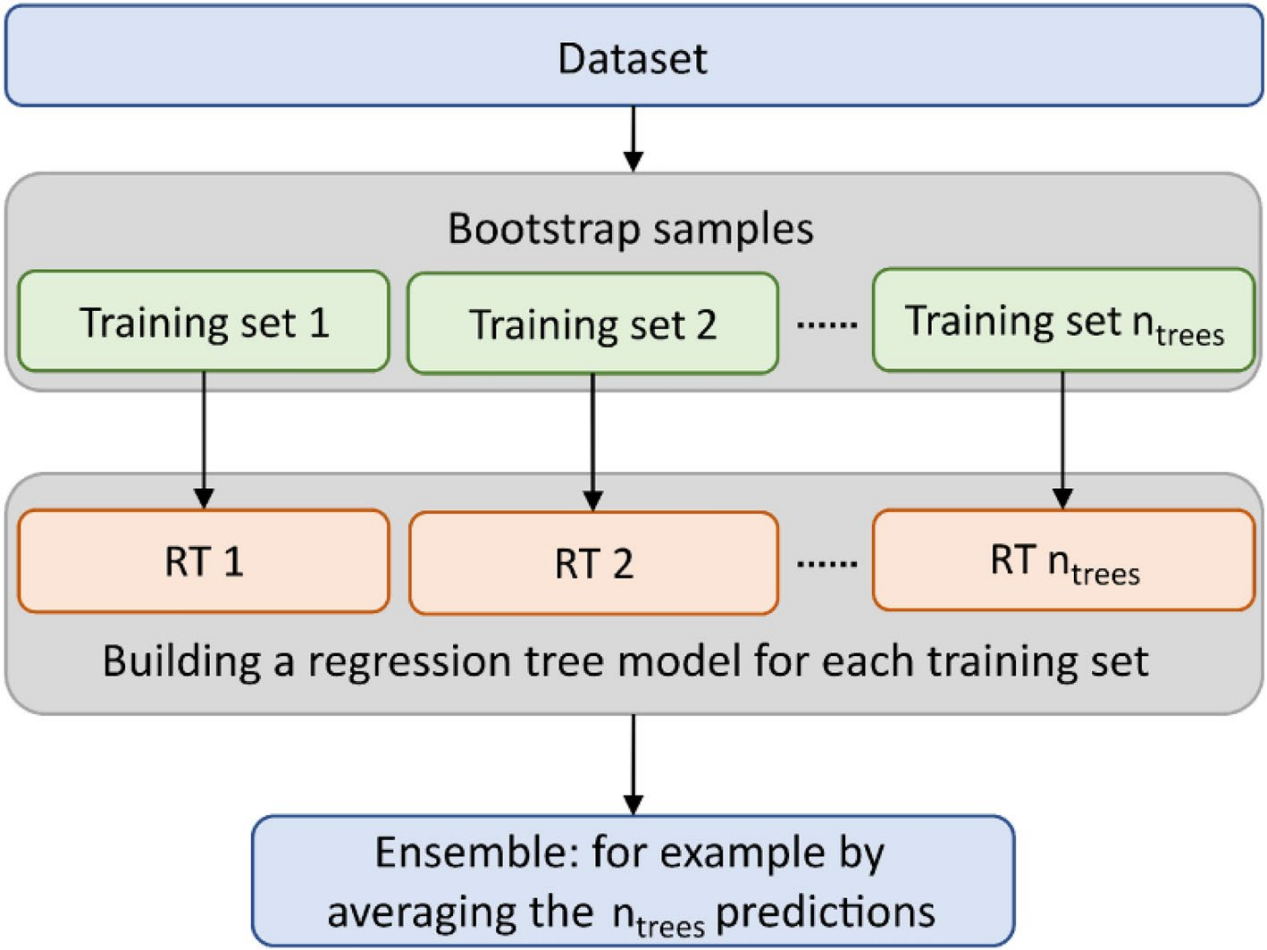
Schematic diagram of random forest algorithm^35^.

Each training subset constructs its own decision tree separately, with the decision tree construction comprising two processes: node splitting and random selection of feature variables. Node splitting is based on splitting rules that compare information attributes and select the attributes with the best comparison results to generate subtrees for growing the decision tree. As depicted in Fig. 2, random feature variable generation is commonly used for the random selection of input variables and information attributes for node splitting. Random selection of training subsets and node attributes ensures the randomness of the random forest to prevent the model from falling into the dilemma of overfitting and local over-optimization. Finally, the average of *n* decision tree regression prediction results is chosen as the final prediction value.

### Support vector regression model

**Figure 3:**
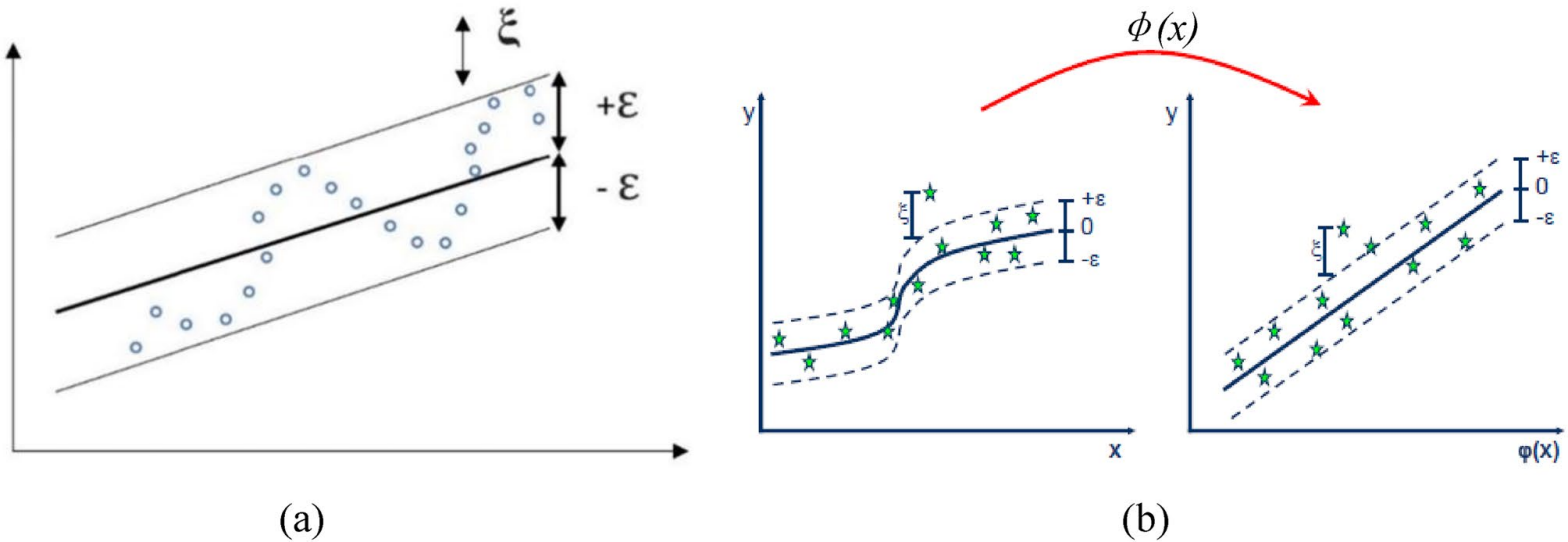
The schematic diagram of SVR.

**Figure 4:**
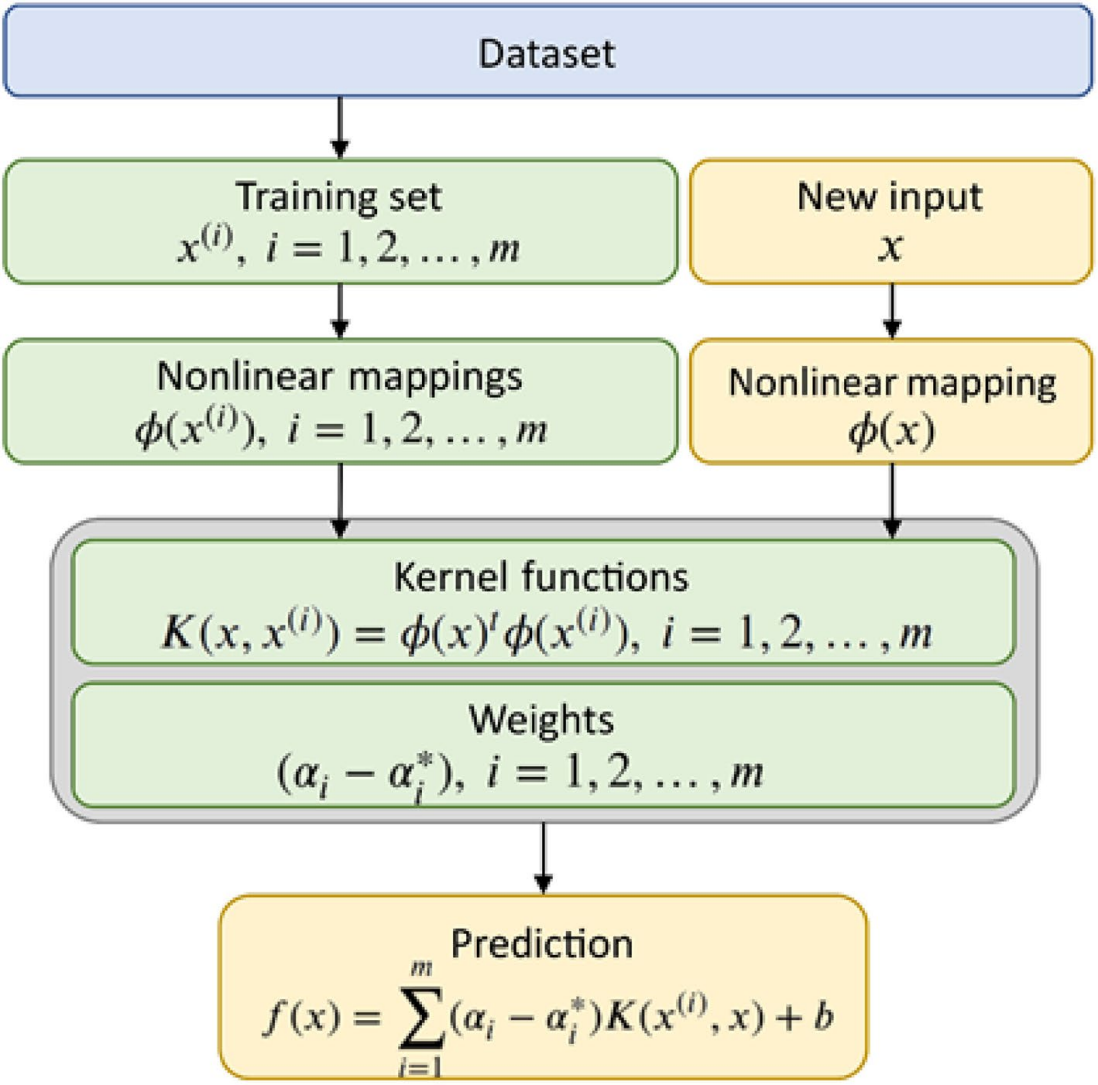
Algorithmic procedure of the SVR^35^.

Support vector regression (SVR) is based on the idea of structural risk minimization and is known for its good performance and predictability when dealing with situations involving small samples, nonlinearities, and large dimensions, as illustrated in Fig. 3. The basic concept behind SVR is to use nonlinearity to map the original data *x* to a high-dimensional feature space, where the linear regression problem can be solved. The regression function of SVR is shown below.

1 $$ f(x) = w \cdot \phi (x) + b $$

where *w* is the weight vector, *b* is the bias, and the following functions can be used to determine *w* and* b*.

2 $$ \left\{ \begin{gathered} {\text{Minimize}}\frac{1}{2}w^{T} + c\sum\nolimits_{i = 1}^{n} {(\xi_{i} + \xi_{i}^{*} )} \hfill \\ y_{i} - w^{T} \phi (x) - b \le \varepsilon + \xi_{i} \hfill \\ w^{T} \phi (x) + b - y_{i} \le \varepsilon + \xi_{i}^{*} \hfill \\ \xi_{i} ,\xi_{i}^{*} \ge 0 \hfill \\ \end{gathered} \right. $$

where *c* is the penalty parameter, $\xi_{i}$ and $\xi_{i}^{*}$ are slack variables, and $\varepsilon$ is the insensitive range. There are various options for the kernel function, and the typical RBF kernel function is utilized in this study. The calculation process of SVR can be represented by the flow chart in Fig. 4.

For regression modeling, the interplay between two hyper-parameters (c and g) has the greatest impact on model accuracy^36^. To address this issue, the grid search (GS) method is introduced, which is widely adopted due to its ease of use and simplicity^37^.

### Support vector regression with grid search optimization

GS's fundamental tenet is to first define the parameter area to be searched, split the region into a grid, and then examine all possible parameter combinations at each intersection point in the grid. All of the grid's intersections represent parameter combinations (*c, g*) that must be searched, and all of the hyperparameter combinations must be taken. Cross-validation is used to verify the prediction accuracy related to each set of data in order to get the best (*c,g*). The sets of (*c,g*) with the best accuracy are chosen as the model's core components. The basic steps of grid parameter optimization search are as follows.

1. Establish the coordinate grid: take x = [−a, a],y = [−b, b], step size *L*, and take the grid points of parameters as c = 2^x^, g = 2^y^.
2. Use k-fold cross-validation to find the regression accuracy: select the training data and divide them into k copies that are uniformly disjoint, select k − 1 of them for model building, and leave the remaining one for validating the model. A set (c,g) in the parameter grid is selected and the prediction accuracy of the test data corresponding to this set (c,g) is recorded. Repeat the preceding processes k times to get k models, then run each model on a different set of test data to get k prediction accuracies. Finally, take the average of these accuracies to get the final corresponding accuracy of the group of parameters.
3. Iterate the coordinate grid: find the final accuracy of all parameter combinations and rank them from largest to smallest, and select the top group as the final (c,g) combination of the model.

**Figure 5:**
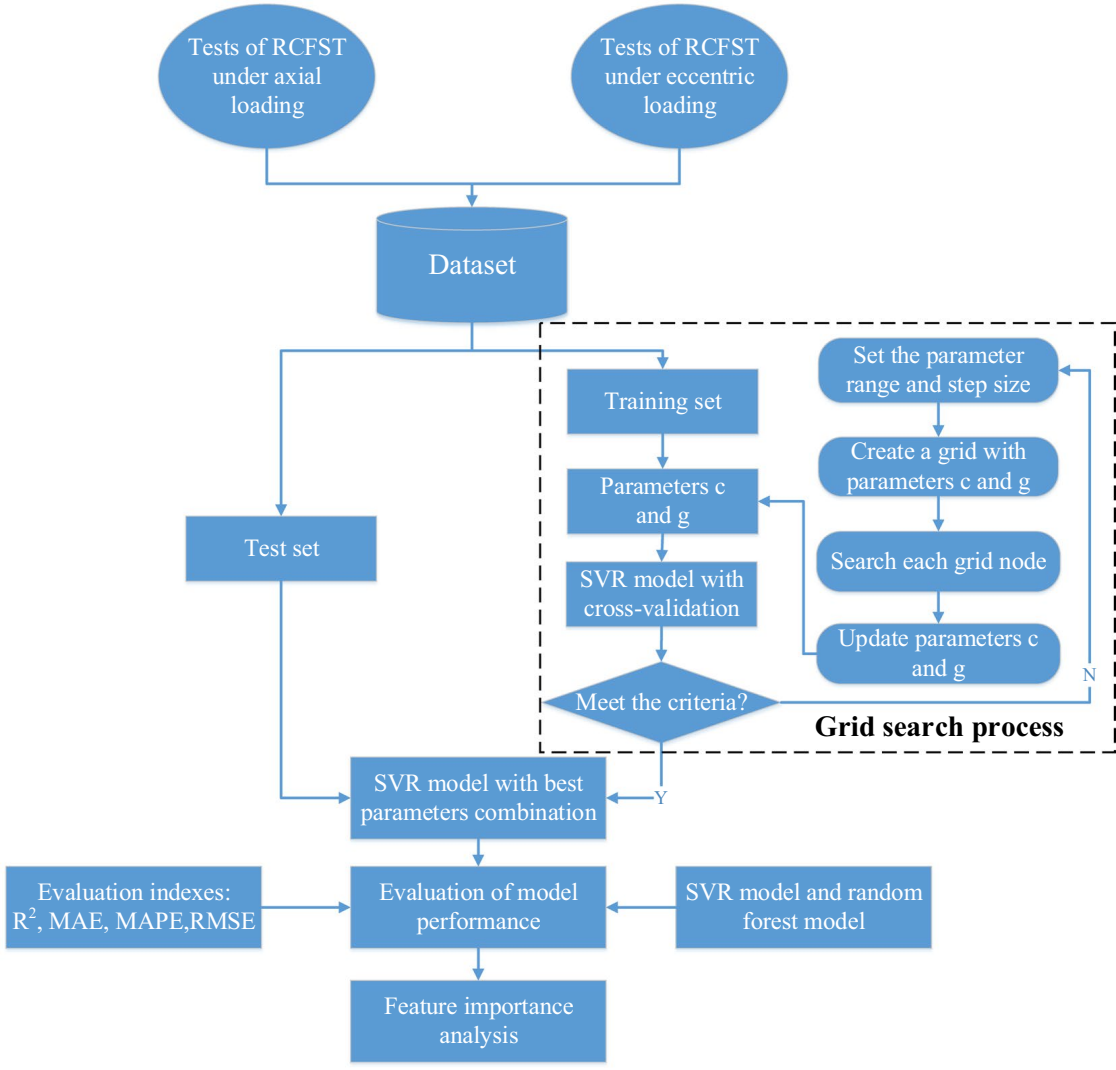
The framework of this paper.

Based on the results of parameter optimization and cross-validation, the best combination of hyper-parameters values is selected to make the system perform best, and the test dataset prediction is implemented using SVR model with optimal parameters. The framework of this paper is shown in Fig. 5.

## Dataset description

**Figure 6:**
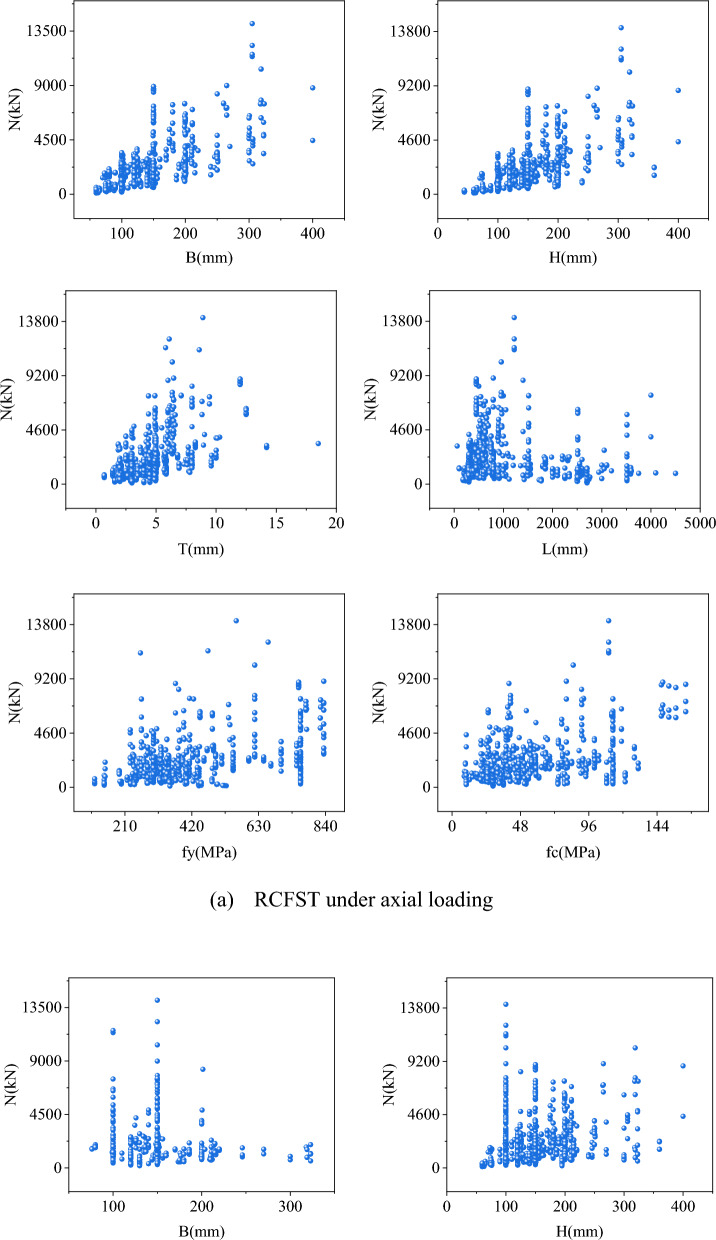

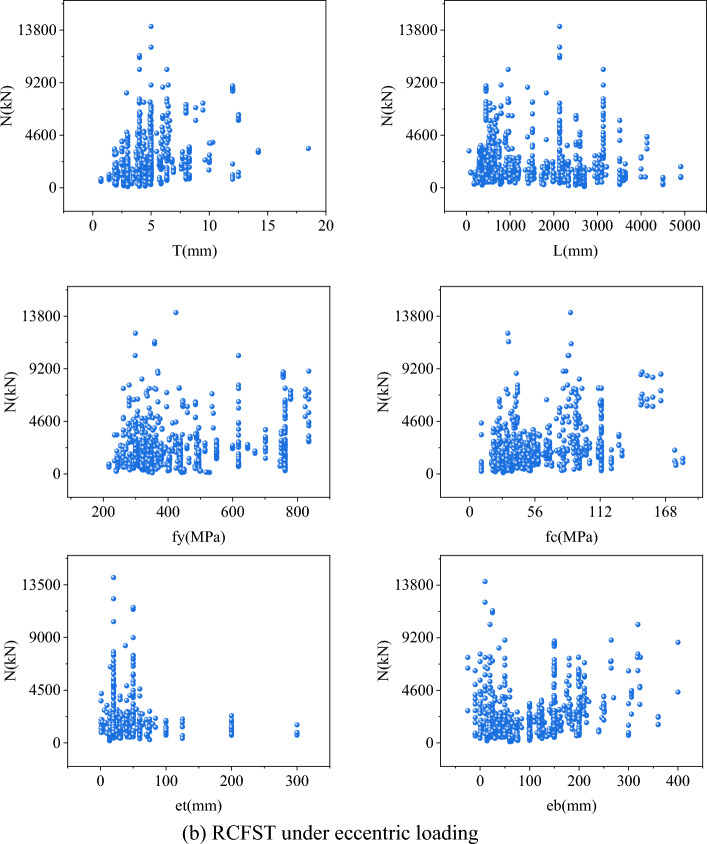
The range distribution of all variables.

To construct a precise strength model for the CCFST column, a comprehensive experimental database is essential. Two datasets, comprising 1003 tests on RCFST columns under axial loading (Dataset 1) and 401 tests on RCFST columns under eccentric loading (Dataset 2), were collected from an open-public dataset^38^. A description of the experimental conditions and more detailed experimental situations for each sample in the data set can be found in Reference^39^ and will not be repeated here. The ranges and statistical characteristics of these datasets are illustrated in Fig. 6 and Table 1, respectively. It is noteworthy that the distribution of maximum bearing capacity exhibits significant variations, which may pose a challenge to accurately predict the outcomes.

**Table 1:** The data set's statistical findings.

Data set	Size	Variable	Unit	Min	Max	Median	Mean	SD	Kurtosis	Skewness
1	1003	*B*	mm	60	400	125	141.59	55.34	2.12	1.39
		*H*	mm	44	400	149.92	153.42	54.71	2.09	1.16
		*T*	mm	0.7	18.5	4.38	4.57	2.23	3.76	1.48
		*L*	mm	60	4500	570	949.63	864.16	1.83	1.64
		*f*_*y*_	MPa	115	835	342	405.82	171.62	0.32	1.17
		*f*_*c*_	MPa	8.52	164.1	44.9	55.07	33.23	0.83	1.17
		*N*	kN	105.4	14,116	1697	2238.31	1919.5	4.99	2.03
2	401	*B*	mm	76.2	323	150	150.52	46.19	2.5	1.28
		*H*	mm	76.2	323	150	154.56	49.57	1.58	1.12
		*T*	mm	1.9	12.5	4.18	4.51	1.65	5.97	1.96
		*L*	mm	330	4910	1800	1776.09	1118.3	−0.59	0.5
		*f*_*y*_	MPa	242	761	340	390.74	126.2	1.54	1.48
		*f*_*c*_	MPa	18.76	183	46.8	57.18	30.64	1.9	1.18
		*et*	mm	0.9	300	30	43.87	45.32	12.22	3.18
		*eb*	mm	−25	300	30	40.79	47.01	11	2.95
		*N*	kN	156.35	7136	981.6	1250.12	1005.45	8.5	2.36

**Figure 7:**
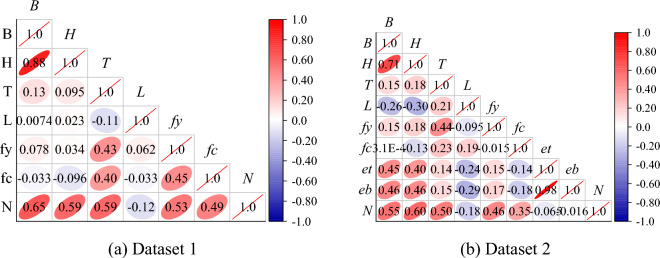
Pearson correlation coefficient of variables.

Also, it can be observed from Fig. 7 that Pearson linear correlations were computed and plotted between the input and output variables in the two data sets. It can be seen from Fig. 7 that the three variables with the strongest linear correlation with the compressive strength of RCFST are *B*, *H*, and *T*, which are all geometric properties. The correlation coefficients of these three variables were 0.65, 0.56, and 0.56 for dataset 1 and 0.55, 0.60, and 0.50 for dataset 2, respectively. And the three of them are positively correlated with the compressive strength, while the *L* is negatively correlated with the compressive strength. Among the multiple variables listed in this paper, all the parameters except *L*, *et*, and *eb* are positive for the bearing capacity of RCFST columns, and *N* increases as these parameters increase. However, the correlation coefficient between input and output variables did not exceed 0.8, indicating that complex nonlinear correlations need to be established between multiple input factors and the output compressive strength to achieve an accurate prediction of compressive strength.

Additionally, the following four metrics were used to evaluate the model's performance: correlation coefficient (R^2^), root mean square error (RMSE), mean absolute error (MAE), and mean absolute percentage error (MAPE). Their definitions are depicted below^40,41^.

3 $$ R^{2} = \frac{{(\sum\limits_{i = 1}^{n} {(T - \overline{T} )} (Y - \overline{Y} ))^{2} }}{{\sum\limits_{i = 1}^{n} {(T - \overline{T} )}^{2} \sum\limits_{i = 1}^{n} {(Y - \overline{Y} )^{2} } }} $$

4 $$ RMSE = \sqrt {\frac{1}{n}\sum\limits_{i = 1}^{n} {(T - Y)^{2} } } $$

5 $$ MAE{ = }\frac{1}{n}\sum\limits_{i = 1}^{n} {(T - Y)} $$

6 $$ MAPE{ = }\frac{100}{n}\sum\limits_{i = 1}^{n} {\left| {\frac{T - Y}{Y}} \right|} $$

where T and Y are the experimental and predicted results, respectively, while $\overline{T}$ and $\overline{Y}$ are the mean values.

## Results and analysis

### Optimization of optimal hyper-parameter combination

**Figure 8:**
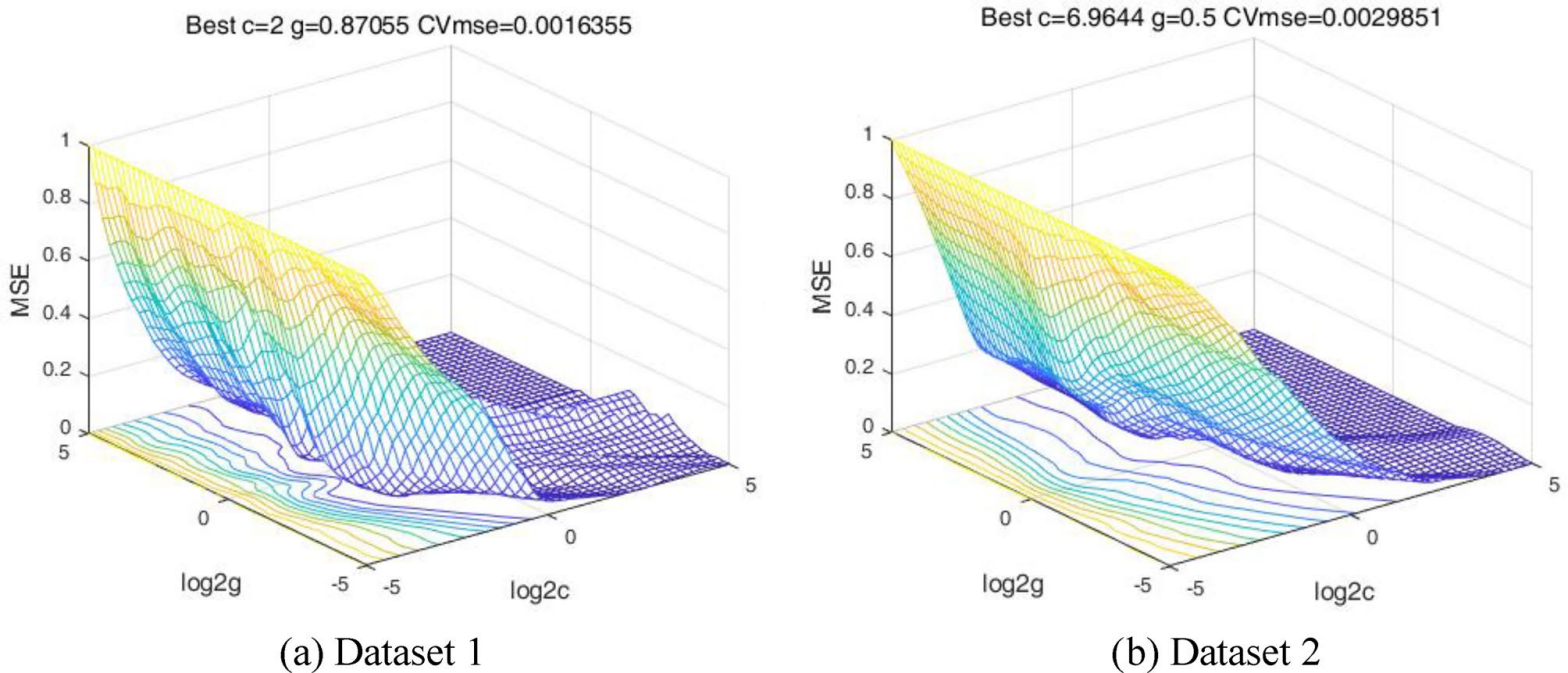
Optimal hyper-parameter combination search using grid search and cross-validation.

The training set and the data set were chosen randomly for each case, with a ratio of 80%:20% between the two. The cross-validation and GS method were used to explore the optimal hyper-parameter combination. The evolution of the mean square error (MSE) and the determination of the optimal parameters during the training in the search range^2–5,25^ are shown in Fig. 8. For dataset 1, the best validation performance of the model is achieved when *c* = 2, *g* = 0.87055, and for dataset 2, when *c* = 6.9644, *g* = 0.5. Then, these two sets of hyper-parameter combinations will be used for the model building of the two data sets respectively.

### Model prediction outcomes comparison

**Figure 9:**
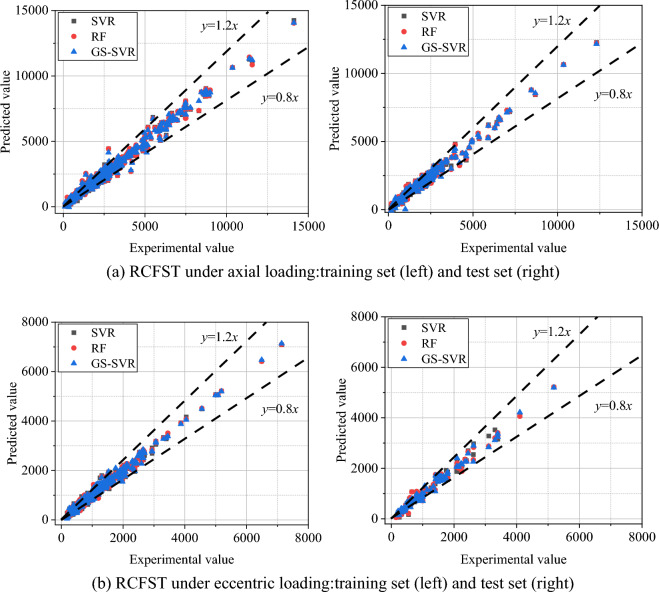
Correlation between expected results and actual values.

Random forest and the original SVR model were also utilized on the same training and test sets for comparison to evaluate the validity and reliability of the proposed models. Figure 9 shows the relationship between the experimental data under various scenarios and the forecasted results of the three models. As seen, for both the training and test sets, the scatter between the three machine learning models' outcomes and actual values is primarily within ± 20%. Unfortunately, Fig. 9 makes it challenging to compare the three models. The error metrics between the predicted outcomes and the actual values of the various models are listed in Table 2 for easy comparison.

**Table 2:** Evaluation indicators for the three models' predictions.

Model	Evaluation indices	Dataset 1		Dataset 2	
		Training	Test	Training	Test
SVR	R^2^	0.981	0.980	0.985	0.978
	MAE	188.123	194.715	94.504	116.753
	RMSE	266.234	262.589	124.929	147.148
	MAPE(%)	12.127	13.837	11.260	13.595
RF	R^2^	0.985	0.980	0.986	0.979
	MAE	164.995	195.491	89.305	108.293
	RMSE	239.469	259.248	119.205	141.457
	MAPE(%)	10.295	13.400	10.794	13.440
GS-SVR	R^2^	0.988	0.983	0.987	0.984
	MAE	144.931	177.062	85.270	93.234
	RMSE	213.687	240.963	114.183	124.924
	MAPE(%)	9.420	12.209	10.066	10.032

The correlations between the predicted and actual values in the hybrid model proposed in this research are 0.983 and 0.984 for two different datasets, respectively, which are higher than those in the two standard machine learning models, RF and SVR. Among the three models, the other three error indicators are the lowest. The results obtained for the axial loading dataset with R^2^ of 0.983, MAE of 177.062, RMSE of 240.963, and MAPE of 12.209%, and for the eccentric loading dataset with R^2^ of 0.984, MAE of 93.234, RMSE of 124.924, and MAPE of 10.032% show that GS-SVR is the best model for predicting the compressive strength of RCFST columns under axial and eccentric loadings.

**Figure 10:**
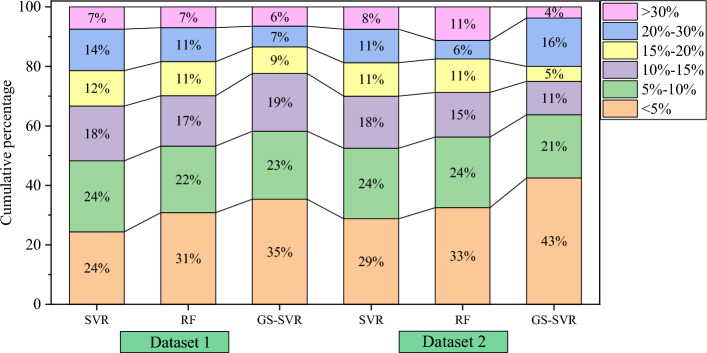
Prediction error distribution of the test set.

**Figure 11:**
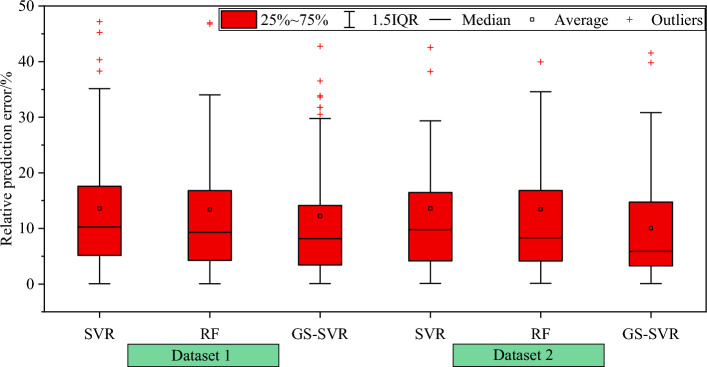
Box plot of prediction error distribution of test set.

Figure 10 offers a comprehensive overview of the prediction error distribution among the models in the test dataset. The findings reveal that, across all three machine learning models, approximately 50% of the test sets exhibit a relative prediction error of 10% or less, while 80% of the test sets display a relative error distribution within the 20% range. Moving to Fig. 11, it presents the prediction error statistics for the test set across various operating conditions for each model. The optimized hybrid model demonstrates an average relative prediction error of 12.209% and 10.032% for the test set under the two different working conditions, respectively. These average relative errors are notably smaller than those of the corresponding SVR and random forest models, with all relative errors falling within the 15% threshold, meeting the requirements for engineering applications.

**Figure 12:**
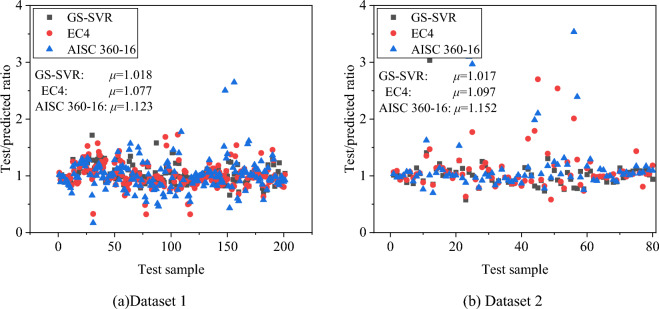
Ratio of experimental values to predicted values for different models.

To further evaluate the performance of the proposed model, two design criteria, AISC 360–16 and Eurocode 4 (EC4), were used to make predictions on the test set and the ratio between the experimental and predicted values of the different models was calculated as shown in Fig. 12. From the mean values *μ* presented in Fig. 12, the ratio between the actual and predicted values in the GS-SVR model is closer to 1, indicating that the predictions are more accurate.

### Input feature analysis

**Figure 13:**
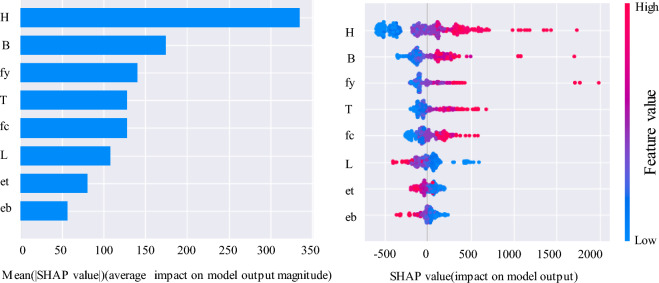
SHAP feature importance and summary plot for RCFST column under eccentric loading.

In addition to accurate load-bearing capacity predictions, the analysis of the importance of design parameters is also a critical step in the design of RCFST columns. This is because adjusting design parameters in order of importance, from high to low, can save time and costs. This section introduces Shap analysis to discuss the impact of various parameters on the output results, as shown in Fig. 13. The factors that have the greatest impact on the load-bearing capacity of the column are, in descending order of importance, H, followed by B, and then fy, T, fc, and L. The eccentricities et and eb have the least impact. Additionally, Fig. 13 also demonstrates whether these impacts are positive or negative. It can be observed that the top five parameters in terms of importance have a positive impact on compressive strength, while length and eccentricity have a negative impact. These influences are extremely helpful in the design of RCFST columns. Designers can adjust the design values of various parameters based on the impact of these design parameters to achieve the desired design objectives.

## Conclusions

This study proposes an optimal hybrid model to accurately predict the strength of RCFST columns under both axial and eccentric loads, shedding light on the complex mechanical behavior of RCFST. The proposed model considers the intricate interactions between geometry, material properties, and compressive strength for various loading scenarios.

For two different test sets, the suggested hybrid model exhibits average relative prediction errors of 12.209% and 10.032%, respectively. These errors are smaller than those of the traditional SVR and random forest models, and all relative errors are under 15%, indicating a high degree of prediction accuracy. Moreover, the proposed hybrid model has certain superiorities over the traditional design codes. Therefore, the optimal hybrid model can serve as a reliable alternative to commonly used design codes for predicting the compressive strength of RCFST columns, which can partially replace laboratory tests to save resources and assist in the design of RCFST columns.

Among the input parameters listed in this paper, the cross-sectional dimensions of the steel tube concrete are the most influential on its compressive strength. In the design of concrete-filled steel tube columns, attention should be given to the width and height of the RCFST column. Parameters et, eb, and L have a negative effect on compressive strength, while other geometric parameters and material properties lead to an increase in compressive strength with an increase in their design values.

The implementation of the proposed model in this paper is on a specific dataset. The applicability and generalizability to other similar datasets need to be further investigated. Also, taking more factors affecting the bearing capacity into account as variables within the model is a focus for future work.

## Data Availability

The datasets used and/or analyzed during the current study available from the corresponding author on reasonable request.
